# Subtle Structural Differences Affect the Inhibitory Potency of RGD-Containing Cyclic Peptide Inhibitors Targeting SPSB Proteins

**DOI:** 10.3390/ijms25126764

**Published:** 2024-06-20

**Authors:** Kefa Li, Yanhong Luo, Weiwei Hu, Jinjin Yang, Danting Zhang, Huan Wei, Tingting You, Hai-Shu Lin, Zhihe Kuang

**Affiliations:** 1 Department of Cell Biology, College of Life Science and Technology, Jinan University, Guangzhou 510632, China; 2 Guangdong Provincial Key Laboratory of Bioengineering Medicine, Guangzhou 510632, China; 3 Guangdong Provincial Biotechnology Drug & Engineering Technology Research Center, Guangzhou 510632, China; 4 National Engineering Research Center of Genetic Medicine, Guangzhou 510632, China; 5 College of Pharmacy, Shenzhen Technology University, Shenzhen 518118, China

**Keywords:** SPRY domain-containing SOCS box protein, inducible nitric oxide synthase, cyclic peptide, inhibitor, crystal structure

## Abstract

The SPRY domain-containing SOCS box proteins SPSB1, SPSB2, and SPSB4 utilize their SPRY/B30.2 domain to interact with a short region in the N-terminus of inducible nitric oxide synthase (iNOS), and recruit an E3 ubiquitin ligase complex to polyubiquitinate iNOS, resulting in the proteasomal degradation of iNOS. Inhibitors that can disrupt the endogenous SPSB-iNOS interactions could be used to augment cellular NO production, and may have antimicrobial and anticancer activities. We previously reported the rational design of a cyclic peptide inhibitor, cR8, cyclo(RGDINNNV), which bound to SPSB2 with moderate affinity. We, therefore, sought to develop SPSB inhibitors with higher affinity. Here, we show that cyclic peptides cR7, cyclo(RGDINNN), and cR9, cyclo(RGDINNNVE), have ~6.5-fold and ~2-fold, respectively, higher SPSB2-bindng affinities than cR8. We determined high-resolution crystal structures of the SPSB2-cR7 and SPSB2-cR9 complexes, which enabled a good understanding of the structure–activity relationships for these cyclic peptide inhibitors. Moreover, we show that these cyclic peptides displace full-length iNOS from SPSB2, SPSB1, and SPSB4, and that their inhibitory potencies correlate well with their SPSB2-binding affinities. The strongest inhibition was observed for cR7 against all three iNOS-binding SPSB proteins.

## 1. Introduction

The SPRY domain-containing SOCS box proteins SPSB1, SPSB2, and SPSB4 are E3 ubiquitin ligases that target inducible nitric oxide synthase (iNOS) for ubiquitination and proteasomal degradation [1,2]. On the other hand, it was recently shown that SPSB3 targets the nuclear cyclic GMP-AMP synthase (cGAS) for degradation [3]. SPSB protein consist of a central SPRY/B30.2 domain and a C-terminal SOCS box [4]. Kuang et al. discovered that SPSB2 utilizes the SPRY/B30.2 domain to interact with a short region in the N-terminus of iNOS, and recruits an E3 ubiquitin ligase complex to polyubiquitinate iNOS, resulting in the proteasomal degradation of iNOS [1]. SPSB2 is, therefore, a negative regulator of iNOS that modulates the lifetime of this enzyme, and thereby controls cellular nitric oxide (NO) production. SPSB1 and SPSB4, but not SPSB3, also bind iNOS via their SPRY/B30.2 domains [1]. It was then shown that SPSB1 and SPSB4 also regulate the proteasomal degradation of iNOS [5,6,7], and SPSB2 is essential for the ubiquitination and clearance of iNOS [8].

iNOS is one of three nitric oxide synthases that catalyze the formation of NO from L-arginine, and it generates relatively high levels of endogenous NO compared to the endothelial and neuronal NOSs (eNOS and nNOS) [9]. NO produced by iNOS is a major source of reactive nitrogen species that plays important roles in host defense against invading pathogens and in some other biological processes [9,10,11]. Since SPSB2-deficient macrophages had prolonged iNOS activity, increased NO production, and enhanced killing of *Leishmania major* parasites, inhibitors that can disrupt the endogenous SPSB-iNOS interactions could be used to augment cellular NO production, and may have antimicrobial and anticancer activities [1]. Indeed, a good number of NO-generating agents have been designed and tested on cancers, many of which killed cancer cells and also sensitized cancers to other cytotoxic drugs [12,13,14]. Site-directed delivery is often employed for such NO-generating agents, as unwanted high-concentration NO is harmful to normal tissues [12,13,14]. Notably, RGD peptides have been widely used to deliver both therapeutic and diagnostic agents to the α_v_β_3_ integrin-expressing cancers [15,16,17,18].

We previously reported the rational design of a cyclic peptide inhibitor (cR8, sequence cyclo(RGDINNNV)) of SPSB2-iNOS interaction, based on a conserved DINNNV region within the N-terminus of iNOS, mediating its interaction with SPSB2 [19]. cR8 was different from other recently reported cyclic peptide [20,21,22,23,24] and peptidomimetic [25] SPSB2 inhibitors in that, in addition to the SPSB2 binding stretch, cR8 possessed an RGD motif for site-directed delivery. It is of importance to deliver anti-cancer NO-generating agents to the cancer sites, so as to avoid unwanted side effects [12,13,14]. The RGD motif may also facilitate the cellular internalization of the RGD-coupled cargos [15,16,17,18]. We demonstrated that cR8 bound to α_v_β_3_ integrin-expressing cells using nuclear magnetic resonance experiments [19].

**Figure 1:**
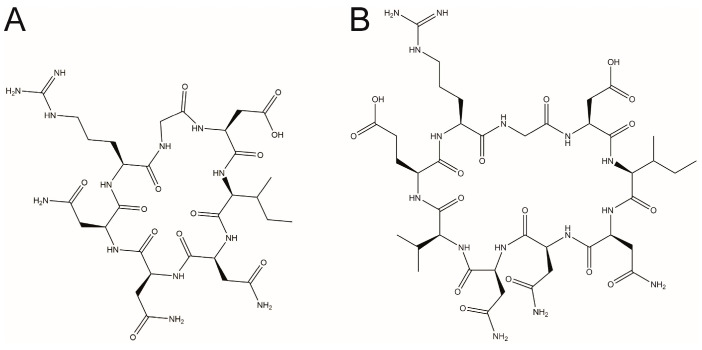
Chemical structures of the rational designed RGD-containing cyclic peptides (**A**) cR7 and (**B**) cR9.

cR8 bound to SPSB2 with a moderate affinity (Kd 671 ± 109 nM), as determined by isothermal titration calorimetry (ITC) [19]. We, therefore, sought to develop SPSB inhibitors with higher affinity. One strategy was to vary the length of the cyclic peptide, while retaining both the SPSB2 binding stretch and the RGD motif. Two novel cyclic peptides, cR7 (sequence cyclo(RGDINNN)) and cR9 (sequence cyclo(RGDINNNVE)), were thus designed, in which the iNOS binding stretch was shortened or extended by one residue compared to cR8. Because the third Asn residue within the DINNN stretch (Asn27 in human iNOS) is the most critical SPSB2-binding residue, the chimeric cyclic peptide could not be further shortened beyond cR7. Chemical structures of cR7 and cR9 are shown in Figure 1.

In this study, we have determined the SPSB2-binding affinities of cR7 and cR9. We then solved the crystal structures of cR7 and cR9 bound to SPSB2 and revealed the structural differences underlying the affinity differences. We also show that cR7, cR8 and cR9 inhibit the interactions of full-length iNOS with SPSB2, SPSB1, and SPSB4, and that their inhibitory potencies correlate well with their SPSB2-binding affinities. These results provide valuable information regarding the structure–activity relationships of the RGD-containing cyclic peptide SPSB inhibitors.

## 2. Results and Discussion

### 2.1. cR7 and cR9 Have Higher SPSB2-Binding Affinities Compared to cR8

**Figure 2:**
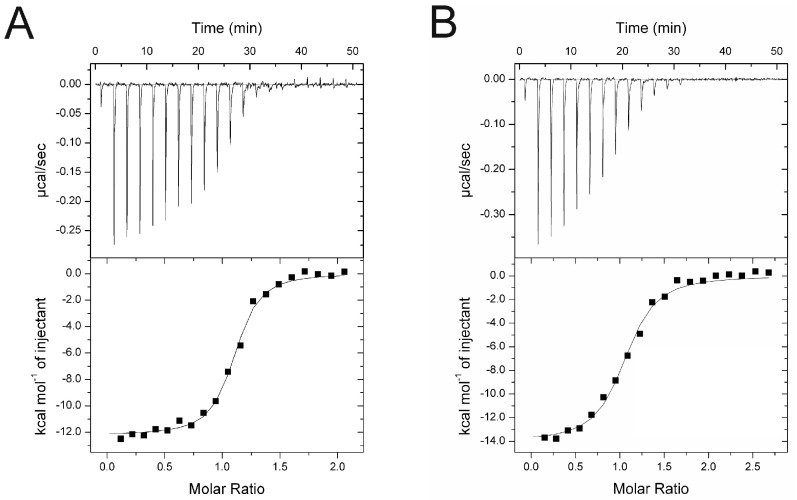
Binding of (**A**) cR7 and (**B**) cR9 to SPSB2 as determined by ITC. Typical raw thermogram data (**upper panels**) and binding isotherm (**lower panels**) are shown. The data were fitted using a single site-binding model.

The binding of cR7 and cR9 to SPSB2 was investigated using ITC (Figure 2). cR7 bound to SPSB2 with a K_d_ of 103 ± 16 nM and a 1:1 binding stoichiometry, whereas cR9 bound to SPSB2 with a K_d_ of 308 ± 51 nM. The SPSB2-binding affinities of cR7 and cR9 were ~6.5-fold and ~2-fold, respectively, higher than those of cR8 reported previously [19].

### 2.2. Crystal Structures of cR7 and cR9 Bound to SPSB2

**Figure 3:**
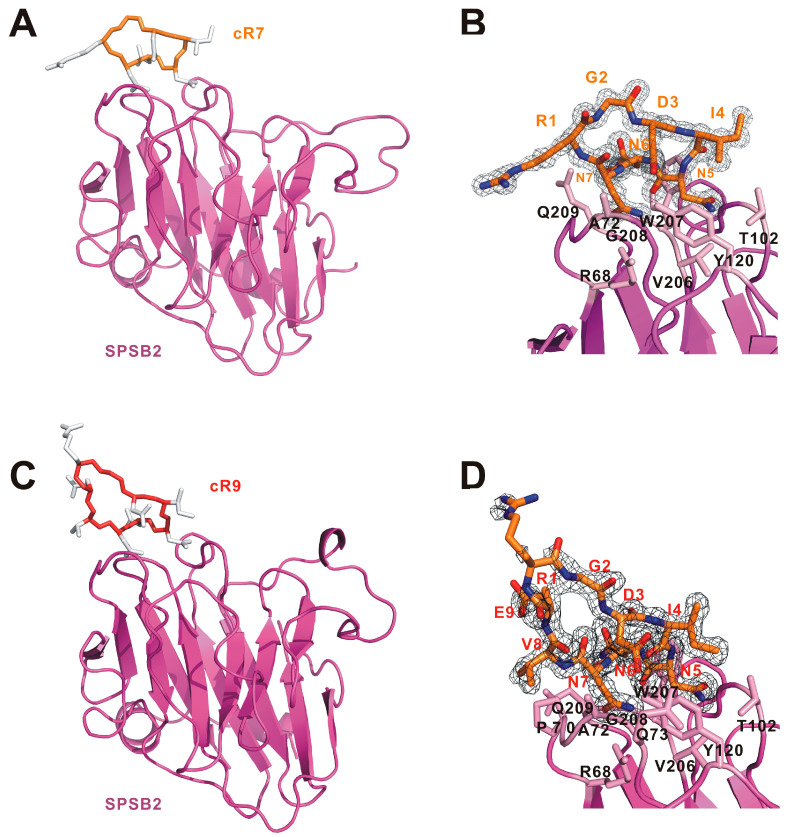
Structural analysis of (**A**,**B**) SPSB2-cR7 and (**C**,**D**) SPSB2-cR9 complexes. (**A**) Overview of the SPSB2-cR7 crystal structure. (**B**) 2Fo-Fc electron density map for cR7. (**C**) Overview of the SPSB2-cR9 crystal structure. (**D**) 2Fo-Fc electron density map for cR9. In (**A**,**C**), SPSB2 molecules are shown as cartoon models and colored pink. cR7 and cR9 are shown as cartoon models with their mainchains colored light orange and dark orange, respectively, and their sidechains colored white. In (**B**,**D**), electron density maps are contoured at 1.0 σ and the sidechains of SPSB2 residues involved in cR7 or cR9 binding are shown and labelled.

In order to understand the structural basis underlying their affinity difference, we have determined the crystal structures of cR7 and cR9 bound to human SPSB2 (Figure 3). Data collection and refinement statistics are listed in Table S1in the Supplementary Data . The SPSB2-cR7 structure was determined at a resolution of 1.23 Å with a final R_free_ value of 0.20, whereas the SPSB2-cR9 structure was determined at 1.61 Å with a final R_free_ of 0.21. In the SPSB2-cR7 structure, clear and continuous electron density is present for the entire cyclic peptide, while in the SPSB2-cR9 structure, electron density is observed for all cR9 residues except for Arg1 and the sidechain of Glu9, indicating disorder of this part of the cyclic peptide that is distal to SPSB2.

**Figure 4:**
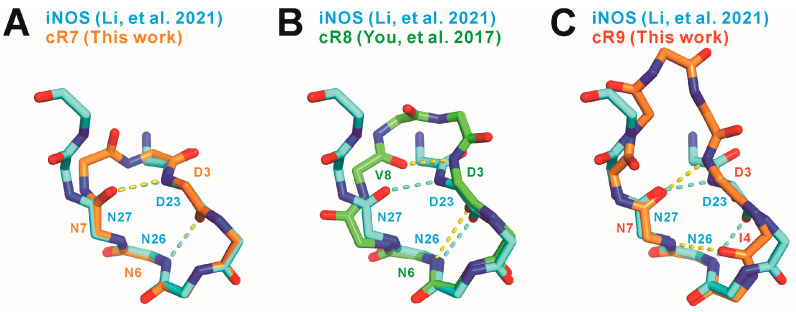
Comparison of the mainchain structures and intra-molecular hydrogen bonds in SPSB2-bound (**A**) cR7, (**B**) cR8 (PDB ID 5XN3 [19]), and (**C**) cR9 to the mainchain structure and intra-molecular hydrogen bonds in SPSB2-bound iNOS N-terminal region (PDB ID 6KEY [2]). cR7, cR8, cR9, and the iNOS N-terminal region are colored light orange, green, dark orange, and cyan, respectively. Nitrogen and oxygen atoms are colored blue and red, respectively. Intra-molecular hydrogen bonds are indicated by yellow dashed lines for cR7, cR8, and cR9, and by cyan dashed lines for the iNOS N-terminal region.

When the SPSB2-bound cR7 structure is superimposed with the SPSB2-bound iNOS N-terminal region structure reported previously [2], we notice that two mainchains superimpose very well and two intra-molecular mainchain hydrogen bonds in the SPSB2-bound iNOS N-terminal region are retained in SPSB2-bound cR7 (Figure 4). These include one hydrogen bond formed between the nitrogen of cR7 Asp3 (equivalent to iNOS Asp23) and the carbonyl oxygen of Asn7 (iNOS Asn27), and another hydrogen bond formed between the carbonyl oxygen of cR7 Asp3 and the nitrogen of cR7 Asn6 (iNOS Asn26). SPSB2-bond cR9 has the Asp3-Asn7 intra-molecular hydrogen bond, but it does not have the Asp3-Asn6 hydrogen bond. It is worth noting that Asn27 and Asp23 in iNOS are the most critical SPSB2-binding residues, as single alanine substitutions of these residues resulted in the largest and the second largest reductions, respectively, in SPSB2-binding affinity [1]. SPSB2-bound cR8 does not have the Asp3-Asn7 inter-molecular mainchain hydrogen bond [19]. The Asp3-Asn7 hydrogen bond in cR7 and cR9 may stabilize their bound structures, resulting in higher SPSB2 binding affinity. On the other hand, the carbonyl group of cR9 Ile4 points in an opposite direction compared to iNOS Ile24, resulting in the formation of a novel Ile4-Asn7 hydrogen bond in cR9 that is absent in iNOS or cR7.

**Figure 5:**
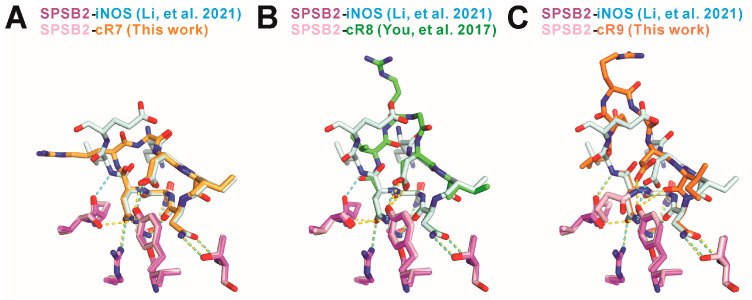
Comparison of the inter-molecular hydrogen bonds in (**A**) SPSB2-cR7, (**B**) SPSB2-cR8 (PDB ID 5XN3, [19]), and (**C**) SPSB2-cR9 complexes to the inter-molecular hydrogen bonds in the SPSB2-iNOS complex (PDB ID 6KEY [2]). cR7, cR8, cR9, and the iNOS N-terminal region are colored light orange, green, dark orange, and cyan, respectively. SPSB2 molecules in a complex with cyclic peptides are colored pink and that in a complex with iNOS N-terminal region is colored purple. Nitrogen and oxygen atoms are colored blue and red, respectively. Inter-molecular hydrogen bonds are indicated by yellow dashed lines for SPSB2-cR7, SPSB2-cR8, and SPSB2-cR9 complexes, and by cyan dashed lines for the SPSB2-iNOS complex.

cR7 and cR9 occupy the shallow iNOS binding pocket formed by SPSB2 residues including Arg68, Pro70-Gln73, Thr102, Tyr120, and Val206-Gln209, which is consistent with the previously reported SPSB2-iNOS [2] and SPSB2-cR8 [19] structures. Interestingly, most of the inter-molecular hydrogen bonds between iNOS and SPSB2 are retained in SPSB2-cR7 and SPSB2-cR9 complexes (Figure 5). These include a hydrogen bond between cR7/9 Asp3 and SPSB2 Tyr120, two hydrogen bonds between cR7/9 Asn5 and SPSB2 Thr102, a hydrogen bond between cR7/9 Asn5 and SPSB2 Gly208, a hydrogen bond between cR7/9 Asn6 and SPSB2 Gly208, as well as three hydrogen bonds between cR7/9 Asn7 and SPSB2 Arg68, Tyr120, and Val206. Interestingly, a mainchain hydrogen bond between iNOS Val28 and SPSB2 Pro70 is absent in the SPSB2-cR7 complex, but is present in the SPSB2-cR9 complex. Furthermore, due to conformational variation, cR9 Asp3 forms a novel sidechain hydrogen bond with SPSB2 Gln73, which is absent in SPSB2-iNOS, SPSB2-cR7, or SPSB2-cR8 complexes.

### 2.3. Inhibitory Potencies of cR7, cR8, and cR9 for SPSB2, SPSB1, and SPSB4

**Figure 6:**
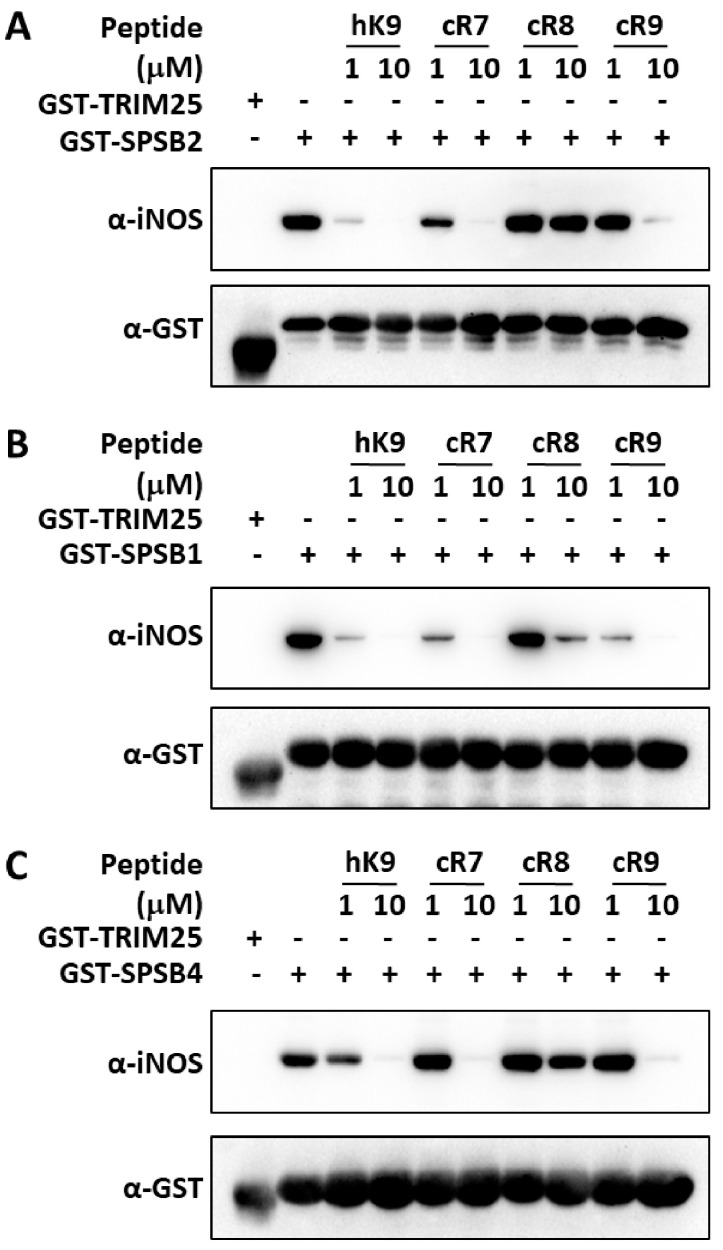
Inhibition of SPSB-iNOS interactions by cR7, cR8, and cR9. Cell lysates from lipopolysaccharide/interferon-γ-stimulated RAW 264.7 cells were incubated with the GST-tagged SPRY domains of (**A**) SPSB2, (**B**) SPSB1, and (**C**) SPSB4 in the absence or presence of cR7, cR8, and cR9. GST-tagged SPRY domain of TRIM25 was used as a negative control. A 9-mer linear peptide (hK9) derived from the iNOS N-terminal region was included as a control. After interaction for 2 h, GST-fusion proteins were recovered via affinity purification using glutathione-sepharose 4B beads. Interaction with full-length iNOS was analyzed by Western blotting with anti-iNOS antibody (upper panels) and equivalent protein input was confirmed by reprobing the membranes with anti-GST antibody (lower panels).

Having established that cR7 and cR9 have higher binding affinities for SPSB2 compared to previously reported cR8, and determined the underlying structural differences, we asked whether the affinity differences correlate with their inhibitory potencies. More importantly, although peptide and peptidomimetic SPSB2 inhibitors have been reported recently [19,20,21,22,23,24,25], to the best of our knowledge, whether they can displace full-length iNOS from the other two SPSB proteins, SPSB1 and SPSB4, has not been determined in the literature. We, therefore, examined the abilities of cR7, cR8, and cR9 to disrupt the interactions of SPSB2, SPSP1, and SPSB4 with full-length iNOS in macrophage cell lysates. Thus, RAW 264.7 cells were stimulated with lipopolysaccharide and interferon-γ to induce iNOS expression. The cells were lysed and the binding of iNOS to SPSB2, SPSB1, and SPSB4 was investigated in the presence of cR7, cR8, or cR9 (Figure 6). The results show that, at a 10 μM peptide concentration, cR7 and cR9 were able to displace full-length iNOS binding to SPSB2, while cR8 was unable to compete with full-length iNOS for binding to SPSB2. Interestingly, the binding of iNOS to SPSB1 appeared to be easier to disrupt, as both cR7 and cR9 at 1 μM, as well as cR8 at 10 μM, can effectively inhibit SPSB1-iNOS interaction. On the other hand, their inhibitory potencies for SPSB4-iNOS interaction were very similar to their potencies for SPSB2-iNOS interaction, as at a 10 μM peptide concentration, cR7 and cR9, but not cR8, were able to displace full-length iNOS binding to SPSB4. Overall, a stronger inhibition was observed for cR7 against all three SPSB proteins.

## 3. Materials and Methods

### 3.1. Synthetic Peptides

The cyclic peptides used in this study, cR7 and cR9, were synthesized by the GL Biochem Ltd. (Shanghai, China) and were head-to-tail amide-cyclized. The purity of the synthetic peptides was ~95% and the observed masses (783.3 Da for cR7 and 1011.8 Da for cR9) were consistent with their theoretical masses (783.8 Da for cR7 and 1012.1 Da for cR9). The cyclic peptide cR8 and the 9-mer linear peptide (hK9, also called iNOS-N peptide in previous publication, sequence Ac-KDINNNVEK-NH_2_, corresponding to the Lys22-Lys30 region of human iNOS) used in the cell lysate inhibition assay have been described previously [2,19].

### 3.2. Protein Expression and Purification

The preparation of the recombinant His_6_-tagged SPRY domain of human SPSB2 (UniProtKB accession number Q99619, residues 22-220) has been previously described [26]. Briefly, SPSB2 protein was initially purified using a Ni-NTA column and the amount of the protein was measured using the Bradford method. Either cR7 or cR9, at 1.5-fold molar excess, was then added to the protein and the SPSB2-cR7 and SPSB2-cR9 complexes were purified by gel filtration using a HiLoad 16/600 Superdex 75 column (GE Healthcare, Marlborough, MA, USA) equilibrated against a buffer containing 50 mM Tris-HCl pH 7.5, 150 mM NaCl, 5 mM DTT. To generate the GST-fusion proteins used in the cell lysate inhibition assay, the SPRY domains of human TRIM25 (UniProtKB Q14258, residues 433-630), SPSB2, SPSB1 (UniProtKB Q96BD6, residues 28-232), and SPSB4 (UniProtKB Q96A44, residues 28-232) were inserted into the pGEX-4T-2 vector (GE Healthcare, Marlborough, MA, USA). Protein expression was induced in an LB medium supplemented with 0.1 mM isopropyl β-d-1-thiogalactopyranoside (IPTG) at 18 °C overnight. Cells were harvested and resuspended in PBS, and then lysed using a cell disruptor (JNBIO, Guangzhou, China). Soluble GST-tagged proteins were purified using a glutathione sepharose 4B column prepared with PBS.

### 3.3. Isothermal Titration Calorimetry

All ITC measurements were performed at 25 °C using iTC200 (Malvern Panalytical, Malvern, UK). SPSB2 was prepared in a buffer containing 50 mM Tris-HCl pH 7.5, 150 mM NaCl, 5 mM DTT, and cR7 and cR9 peptides were prepared in the same buffer from 2.5 mM stocks. ITC measurements were carried out by titrating 100 μM cR7 or cR9 solutions into 10 μM SPSB2. Data analysis was performed using the Origin 6.1 software package provided with the instrument. Parameters were derived using a single binding site model. The dissociation constant (K_d_) was calculated, with the error from the Origin-calculated association constant (K) transferred as the same fraction of the primary value.

### 3.4. Crystallization, Data Collection and Structure Determination

Crystallization was carried out using the hanging drop vapor diffusion method. Crystals of the SPSB2-cR7 complex grew at 20 °C from a solution containing equal volumes of 8.5 mg/mL of a protein solution and reservoir solution containing 0.2 M sodium chloride, 0.1 M Bis-Tris pH 6.5, 25% *w*/*v* polyethylene glycol 3350, whereas SPSB2-cR9 crystals grew from a solution containing equal volumes of 10 mg/mL of a protein solution and reservoir solution containing 0.02 M citric acid, 0.08 M Bis-Tris propane pH 7.6, 14% *w*/*v* polyethylene glycol 3,350. Just prior to data collection, crystals were transferred to a cryoprotectant solution consisting of the reservoir solution supplemented with 25% (*v*/*v*) glycerol. Diffraction data sets were collected using beamline BL19U1 at the Shanghai Synchrotron Radiation Facility (SSRF, Shanghai, China). Structure determination was carried out using the CCP4 7.0 package [27]. Indexing and integration was performed using iMOSFLM [28] and scaling was performed using AIMLESS [29]. Molecular replacement was performed with MOLREP [30] using the SPSB2-cR8 structure (PDB ID 5XN3) [19]. Structure models were built in COOT [31] and refined using REFMAC5 [32]. Structure figures were prepared using the PyMOL molecular graphics system (Schrödinger, New York, NY, USA).

### 3.5. Cell Lysate Inhibition Assay

The assay for the inhibition of iNOS binding to SPSB proteins was conducted as described in the literature [1,24]. RAW 264.7 cells were collected 16 h after treatment with 1 μg/mL of lipopolysaccharide (Beyotime, Shanghai, China) and 100 ng/mL of murine interferon-γ (Sino Biological, Beijing, China). Cells were lysed in a buffer containing 50 mM Tris-HCl pH 7.5, 150 mM NaCl, 5 mM EDTA, 0.2% Triton X-100, 10 mM Na_3_VO_4_, and cOmplete ULTRA protease inhibitors (Roche) on ice for 20 min. The cell lysates were incubated with 2 μg/mL of GST-tagged SPRY domain of TRIM25 (as negative control) or GST-tagged SPRY domains of SPSB2, SPSB1, and SPSB4, in the absence or presence of 1 μM or 10 μM of cyclic peptides for 2 h. A 9-mer linear peptide (hK9) derived from the iNOS N-terminal region was included as a control, as inhibition was observed for a similar linear peptide at 1 to 10 μM of a peptide concentration [1]. The SPSB-iNOS protein complexes were recovered from the lysates via incubation with glutathione sepharose 4B (GE healthcare, Marlborough, MA, USA) for 1 h. Proteins were separated by SDS-PAGE under reducing conditions and transferred onto nitrocellulose membranes (Millipore). Membranes were blocked overnight in 10% (*w*/*v*) skim milk prior to incubation with a monoclonal anti-iNOS primary antibody (BD Biosciences) for 2 h. Antibody binding was visualized with a horseradish peroxidase (HRP)-conjugated sheep anti-mouse IgG antibody (Beyotime) and an enhanced chemiluminescence (ECL) system (Beyotime). To confirm equivalent protein loading, the membranes were re-blotted with an Anti-GST rabbit polyclonal antibody (Sangon, Shanghai, China) for 2 h after stripping off the anti-iNOS and HRP-conjugated sheep anti-mouse IgG antibodies in 0.1 M glycine pH 2.9. Anti-GST antibody binding was then visualized with HRP-conjugated sheep anti-rabbit IgG antibody (Beyotime) and ECL.

## 4. Conclusions

In this study, we found that both the 7-mer (cR7) and the 9-mer (cR9) RGD-containing cyclic peptides have significantly higher SPSB2-binding affinity compared to the 8-mer cyclic peptide cR8 reported previously. In particular, although the SPSB2-binding stretch is shortened by one residue in cR7 compared to cR8, cR7 has a ~6.5-fold increased SPSB2-binding affinity than cR8. We determined high-resolution crystal structures of the SPSB2-cR7 and SPSB2-cR9 complexes. These structures, together with the SPSB2-iNOS and SPSB2-cR8 crystal structures we reported previously, enabled a good understanding of the structure–activity relationships for these cyclic peptide inhibitors. Remarkably, SPSB2-bound cR7 superimposed very well to the SPSB2-bound iNOS N-terminal region and both of the Asp23-Asn26 and Asp23-Asn27 intra-molecular mainchain hydrogen bonds in iNOS were retained in cR7. On the other hand, cR9, like cR8, has small structural variations compared to the SPSB2-bound iNOS N-terminal region and intra-molecular hydrogen bonds are somewhat different. More importantly, we show that these cyclic peptides displace full-length iNOS from SPSB2, SPSB1, and SPSB4 in a more physiological setting, and that their inhibitory potencies correlate well with their SPSB2-binding affinities. The strongest inhibition was observed for cR7 against all three iNOS-binding SPSB proteins.

## Data Availability

The coordinates and structure factors of the SPSB2-cR7 and SPSB2-cR9 complexes have been deposited in the Protein Data Bank with the accession codes 6JWM and 6JWN, respectively.

## Supplementary Materials

The supporting information can be downloaded at: https://www.mdpi.com/article/10.3390/ijms25126764/s1.

## Abbreviations

cGAS	Cyclic GMP-AMP synthase
ELC	Enhanced chemiluminescence
eNOS	Endothelial nitric oxide synthase
HRP	Horseradish peroxidase
iNOS	Inducible nitric oxide synthase
ITC	Isothermal titration calorimetry
nNOS	Neuronal nitric oxide synthase
NO	Nitric oxide
RGD	Arginine-glycine-aspartic acid
SOCS	Suppressor of cytokine signaling
SPSB	SPRY domain-containing SOCS box protein
