# Supplementary material for: Subtle Structural Differences Affect the Inhibitory Potency of RGD-Containing Cyclic Peptide Inhibitors Targeting SPSB Proteins
Source: Int J Mol Sci. 2024 Jun 20;25(12):6764. doi: 10.3390/ijms25126764 (PMC11203437; doi:10.3390/ijms25126764)
Supplement: Supplementary file 1 [file ijms-25-06764-s001.zip › ijms-3030518-supplementary.pdf]

Supporting Information

Table S1. Data collection and refinement statistics

	SPSB2-cR7	SPSB2-cR9
**PDB**	6JWM	6JWN
**Data collection**		
Space group	$P2_12_12_1$	P1
Cell dimensions		
a, *b*, *c* (Å)	40.06, 63.97, 68.84	32.40, 46.65, 61.08
α, β, γ (°)	90, 90, 90	87.69, 74.90, 89.91
Resolution (Å)	63.97-1.23 (1.26-1.24)[a]	59.05-1.61 (1.64-1.61)
Total observations	425130 (21600)	228453 (8711)
Unique reflections	51393 (2610)	42845 (1810)
R_{merge}	0.082 (0.305)	0.129 (0.278)
$I / \sigma(I)$	14.4 (5.8)	7.6 (3.6)
Completeness (%)	99.8 (99.9)	96.3 (80.5)
Redundancy	8.3 (8.3)	5.3 (4.8)
**Refinement**		
Resolution (Å)	46.86-1.23	59.05-1.61
No. reflections	48738	40695
R_{work} / R_{free}[b]	0.18/0.20	0.19/0.21
No. of atoms		
Protein	1524	2978
Peptide	55	142
Water	118	137
B-factors		
Protein	8.77	13.83
Peptide	12.56	22.26
Water	15.98	17.88
R.m.s. deviations		
Bond lengths (Å)	0.016	0.013
Bond angles (°)	1.84	1.65
Ramachandran plot		
Most favored (%)	93.0	90.4
Allowed (%)	7.0	9.6
Disallowed (%)	0	0

[a] Values in parentheses are for highest-resolution shell.

[b] 5% of the reflections were selected for the R_{free} and omitted from the refinement process.
